# Loss of radioactivity in radiocesium-bearing microparticles emitted from the Fukushima Dai-ichi nuclear power plant by heating

**DOI:** 10.1038/s41598-018-28087-5

**Published:** 2018-06-26

**Authors:** Taiga Okumura, Noriko Yamaguchi, Terumi Dohi, Kazuki Iijima, Toshihiro Kogure

**Affiliations:** 1 Department of Earth and Planetary Science, Graduate School of Science, The University of Tokyo, 7-3-1 Hongo, Bunkyo-ku, Tokyo, 113-0033 Japan; 2 Institute for Agro-Environmental Sciences, NARO, 3-1-3 Kannondai, Tsukuba, 305-0864 Japan; 3 Fukushima Environmental Safety Center, Sector of Fukushima Research and Development, Japan Atomic Energy Agency, 10-2 Fukasaku, Miharu-machi, Tamura-gun, Fukushima, 963-7700 Japan

## Abstract

Radiocesium-bearing microparticles (CsPs) substantially made of silicate glass are a novel form of radiocesium emitted from the broken containment vessel of Fukushima Dai-ichi nuclear power plant. CsPs have a potential risk of internal radiation exposure caused by inhalation. Radiation-contaminated waste including CsPs is being burned in incinerators; therefore, this study has investigated the responses of CsPs to heating in air. The radioactivity of CsPs gradually decreased from 600 °C and was almost lost when the temperature reached 1000 °C. The size and spherical morphology of CsPs were almost unchanged after heating, but cesium including radiocesium, potassium and chlorine were lost, probably diffused away from the CsPs. Iron, zinc and tin originally dissolved in the glass matrix were crystallized to oxide nanoparticles inside the CsPs. When the CsPs were heated together with weathered granitic soil that is common in Fukushima, the radiocesium released from CsPs was sorbed by the surrounding soil. From these results, it is expected that the radioactivity of CsPs will be lost when radiation-contaminated waste including CsPs is burned in incinerators.

## Introduction

The accident at the Fukushima Dai-ichi nuclear power plant (FDNPP) in March 2011 caused the release of various radionuclides including radiocesium (^134^Cs and ^137^Cs) which is the major source of the high radiation around Fukushima at present due to its large amount and relatively long half-life (30.1 years for ^137^Cs). Two forms or states of radiocesium are now believed to have been released from FDNPP. One is the radiocesium attached to sulfate aerosols in the atmosphere, which was dissolved into raindrops and fell to the ground^1,2^. Such radiocesium was strongly fixed by specific clay minerals, such as weathered biotite^3,4^.

The second is an insoluble particulate form, which was released directly from the broken reactors. Adachi *et al*.^5^ discovered on an aerosol filter spherical radioactive microparticles of 2.0–2.6 μm in diameter with 0.7–3 Bq of ^137^Cs in a single particle. Hereafter, such radiocesium-bearing microparticles are termed “CsPs” in this study. Abe *et al*.^6^ reported that some CsPs contain several fission products of ^235^U other than radiocesium, as well as Fe and Zn which are also used in nuclear reactors, confirming that CsPs were formed in the reactors. Yamaguchi *et al*.^7^ investigated similar CsPs attached to the cloth covering the ground and in leaves in Fukushima, mainly using transmission electron microscopy (TEM). They reported that the material of CsPs is substantially silicate glass in which Cl, K, Mn, Fe, Zn, Rb, Sn and Cs are dissolved as major constituent elements. Kogure *et al*.^8^ further characterized the inner structure of CsPs using energy-dispersive X-ray spectrometers (EDS) with ultra-high detection efficiency attached to a scanning transmission electron microscope (STEM), to investigate the formation mechanism of CsPs. They determined accurate chemical compositions and elemental distributions in CsPs. Satou *et al*.^9^ reported unshaped CsPs collected from soil in Namie, 20 km northwest from the FDNPP. Furthermore, Yamaguchi *et al*.^10^ discovered several new types of CsPs in which the constituent elements and inner structures were different from those reported in the previous works^8,9,11^. From these studies, our knowledge with respect to CsPs including their structure, composition, and location in the field, has gradually increased. However, their formation mechanism in the reactors, their abundance and distribution in the field, and their lifetime and fate in various environments, etc. are still unclear.

In the radiation-contaminated soil collected in Fukushima, clay minerals 50 μm in size sorbing radiocesium contained 10^−3^ to 10^−2^ Bq of ^137^Cs^12^. However, the radioactivity of ^137^Cs in CsPs is far higher, approaching a few Bq. Accordingly, internal exposure to radiation caused by the inhalation of CsPs arouses concern. Moreover, since the size of CsPs is around a few microns, similar to PM_2.5_, their impact on the human respiratory system is also of concern. Radiation-contaminated waste including CsPs is being burned in incinerators to reduce its volume in Japan; therefore, there is an urgent need to reveal the responses of CsPs to heating.

In the present study, we have made an important discovery that the radioactivity of CsPs was decreased by heating in air at a temperature more than 600 °C and was completely lost if heated to around 1000 °C. Electron microscopic analysis revealed that the morphology of CsPs was unchanged but cesium was released from CsPs by heating. This property of CsPs is completely different from that of soil particles sorbing radiocesium^13^. This means that if radiation-contaminated waste including CsPs is burned in incinerators at a sufficiently high temperature, we no longer need to fear the diffusion of CsPs from ash formed by burning.

## Results

### Loss of radioactivity in CsPs by heating

**Figure 1:**
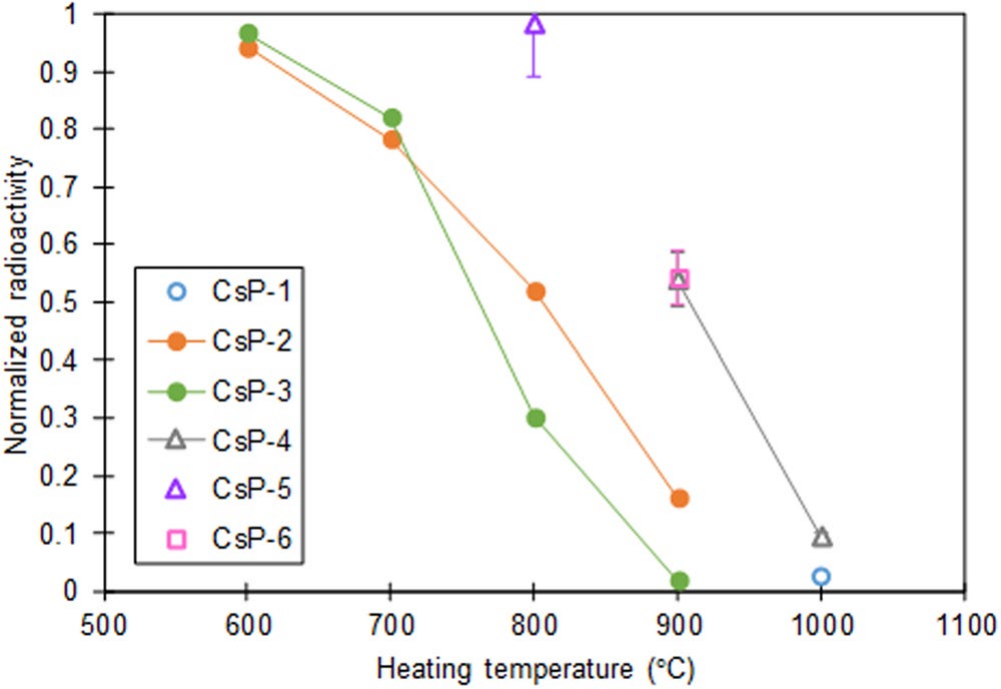
Radioactivity change of CsPs by heating. Radioactivity normalized by that before heating is shown as a function of heating temperature. In each heating operation, CsPs were heated up to the target temperature at 10 °C/min and cooled in air. Points connected by lines mean that heating and cooling were repeated at subsequently increasing target temperatures. Error bars are not shown in CsP-2 and 3 because their radioactivity was determined by IP autoradiography. The radioactivity of other CsPs was measured using a germanium detector.

Six CsPs, named CsP-1 to 6, were heated in a temperature range of 600–1000 °C and their radioactivity was determined before and after heating. The change of radioactivity normalized according to the original radioactivity is shown in Fig. 1. CsP-1 to 3 were put into a hole in a platinum plate using a micro-sampling unit attached to a focused ion beam (FIB) system, and presented for the heating experiments. CsP-4 to 6 were dropped into a platinum pan and presented for the heating experiments. As for CsP-6, soil with a negligible amount of radiocesium was added to the pan to cover the CsP before heating. The radioactivity was measured after each heating operation, in which CsPs were heated up to a target temperature at a rate of 10 °C/min and cooled in air. The data points of CsP-2 to 4 are connected by lines in Fig. 1 because heating and cooling were repeated at target temperatures of 600, 700, 800 and 900 °C for CsP-2 and 3, and of 900 and 1000 °C for CsP-4. The radioactivity of CsPs except CsP-2 and 3 was measured using a germanium detector, whereas that of CsP-2 and 3 was determined by imaging plate (IP) autoradiography^4^. Since radioactivity is calculated from the signal intensity in readout images of IPs in IP autoradiography, the error was not evaluated for CsP-2 and 3. As a result of the heating experiments, the radioactivity of CsPs decreased at a temperature of more than 600 °C and was almost lost at around 1000 °C although the decrease rates varied. Since the radioactivity of CsPs except CsP-1 to 3 also decreased, the loss of radioactivity originated not from the artifacts of FIB processes for picking up CsPs but from the properties of CsPs themselves.

### Effects of heating on the morphology and composition of CsPs

**Figure 2:**
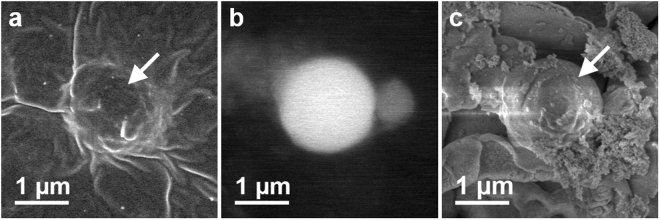
Morphologies of CsP-2 before and after heating. SE (**a**) and BSE (**b**) images of CsP-2 before heating. (**c**) SE image of CsP-2 after heating at 900 °C. White arrows in (**a**) and (**c**) indicate CsP-2.

In order to investigate the effects of heating on the morphology of CsPs, we observed CsPs before heating using scanning electron microscopy (SEM). Figure 2a,b shows secondary electron (SE) and back-scattered electron (BSE) images of CsP-2, on Kapton tape. Although this CsP was almost buried in the glue of Kapton tape, the BSE image indicated that it had a spherical shape, which is typical of CsPs. The CsP was put into a hole made in a platinum plate using FIB and heated as mentioned above. Figure 1c shows the SE image of CsP-2 after heating at 900 °C. The surrounding Kapton tape was burned off and CsP-2 was exposed to view. CsP-2 remained its spherical shape, and its size was also unchanged even after heating. This result indicated that heating at 900 °C did not alter the morphological features of CsPs.

**Figure 3:**
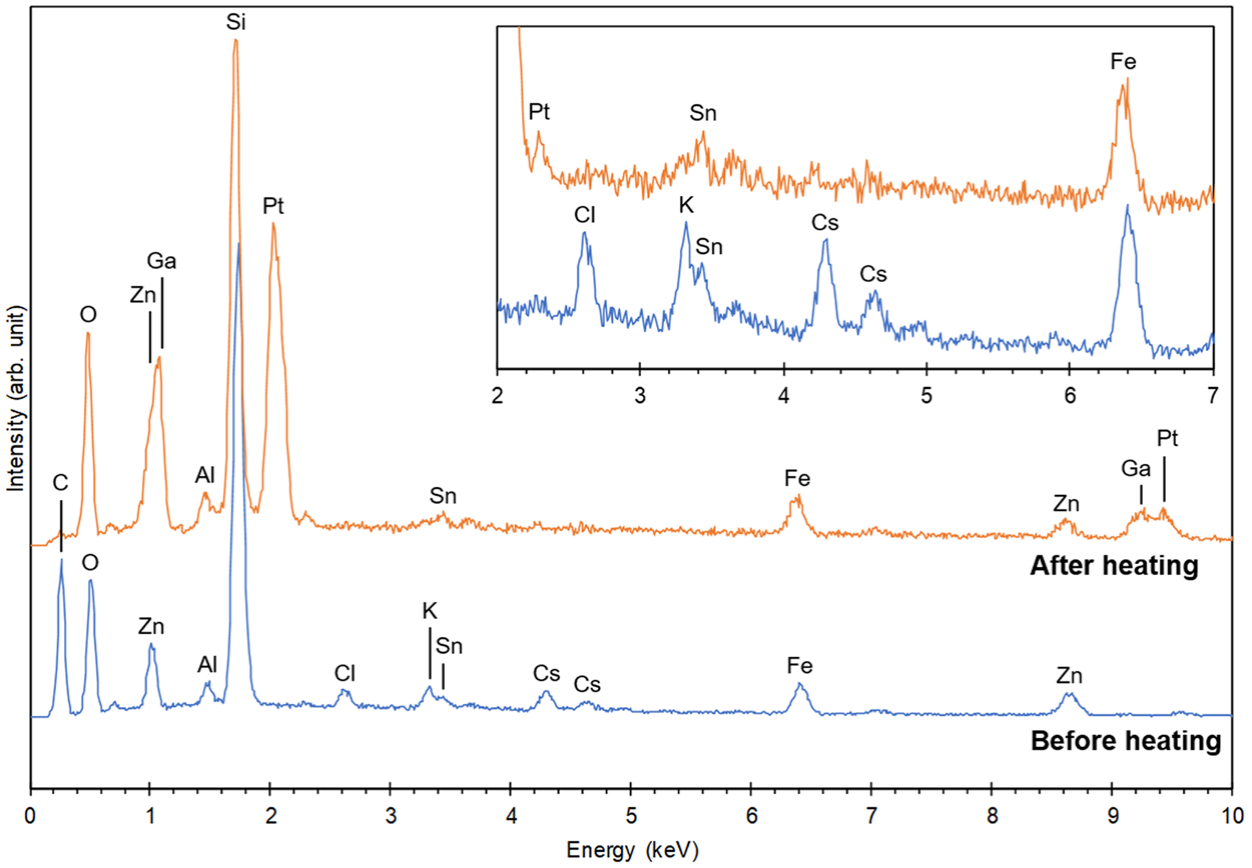
Composition change of CsP-3 by heating. SEM-EDS spectra acquired from CsP-3 before and after heating at 900 °C. The inset shows the enlarged spectra in the range of 2–7 keV.

**Figure 4:**
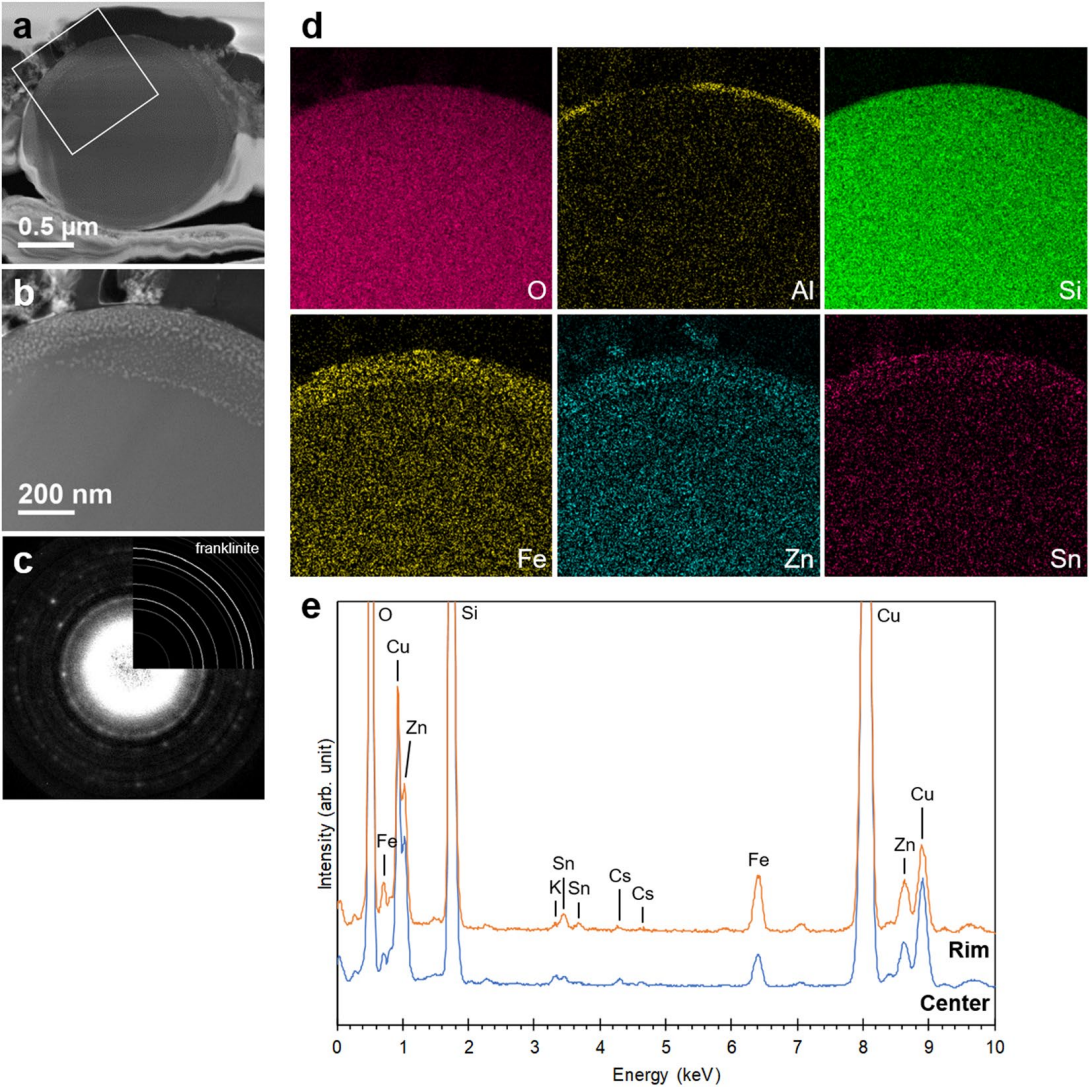
Structures and compositions of CsP-2 after heating at 900°C. (**a**) STEM-ADF image of CsP-2. (**b**) Enlarged view of the white square in (**a**). (**c**) Electron diffraction pattern acquired from the rim of CsP-2, in which many tiny bright spots exist in the STEM-ADF image. The inset shows the calculated Debye-Scherrer pattern of franklinite (ZnFe_2_O_4_). (**d**) Element maps of the same frame as (**b**). (**e**) EDS spectra acquired from the center and rim of CsP-2. The peak intensity is normalized for quantitative comparison.

The effects of heating on the composition of CsPs were also investigated using SEM-EDS (Fig. 3). The spectrum of CsP-3 before heating showed Si and O as the main elements, and Cl, K, Fe, Zn, Sn and Cs as minor elements, which is a typical composition for CsPs^7^. Although Al was also detected in CsP-3, it was probably due to adhering soil particles (see the results of STEM-EDS in Fig. 4). Carbon originated from the Kapton tape. Because Cl, K and Cs disappeared in CsP-3 by heating at 900 °C, these elements may have been released from the CsP mainly as a form of KCl and CsCl. Note that Pt and Ga were detected because of a platinum plate for a container of the CsP and a gallium ion beam in FIB, respectively. Although the compositions of CsP-2 changed similarly to CsP-3, a portion of Cs remained in the CsP even after heating (Supplementary Fig. 1). This is consistent with the result that CsP-2 retained 16% of its original radioactivity after heating, as shown in Fig. 1.

More detailed examinations of heated CsPs were conducted using TEM/STEM. Figure 4a is a STEM-annular dark-field (ADF) image of CsP-2 after heating at 900 °C. In the enlarged view, nanometric crystals identified as franklinite (ZnFe_2_O_4_) by electron diffraction were observed at the rim of the CsP (Fig. 4b,c). The element maps of the CsP were also acquired using STEM-EDS (Fig. 4d), indicating the enrichment of Fe, Zn and Sn in the same region as franklinite. Since such a distribution has not been reported to date, these elements are believed to have migrated within the CsP and to have crystallized as franklinite. Furuki *et al*.^11^ reported the existence of franklinite in original CsPs; however, we suspect that it was caused by irradiation damage because silicate glass is very sensitive to an electron beam^7^. We actually confirmed that Debye-Scherrer rings corresponding to a spinel structure appeared when an intense electron beam was used to irradiate CsPs although a halo corresponding to amorphous materials was initially observed in the electron diffraction pattern. Al existed at the periphery of the CsP, meaning that it did not originate from the CsP but foreign substances such as soil particles, as mentioned above. Cl, K and Cs were not detected in the EDS spectra obtained from the center and rim of the CsP although K and Cs slightly remained in the center region because CsP-2 retained 16% of its original radioactivity after heating (Fig. 4e). These results also suggest that these elements migrated within CsPs and were released from the surface of CsPs.

**Figure 5:**
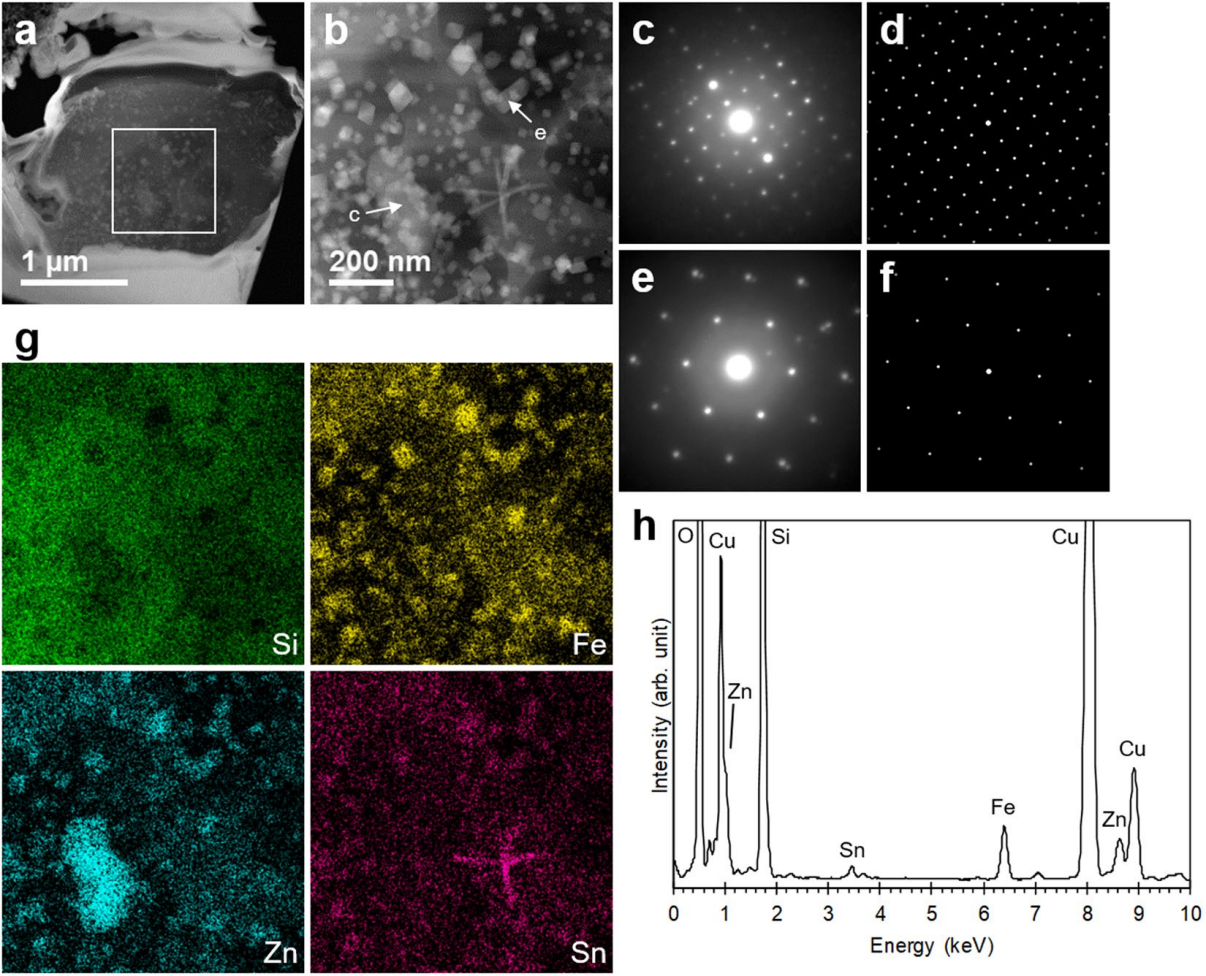
Structures and compositions of CsP-3 after heating at 900°C. (**a**) STEM-ADF image of CsP-3. (**b**) Enlarged view of the white square in (**a**). (**c**) Electron diffraction pattern acquired from the area indicated by c in (**b**). (**d**) Calculated electron diffraction pattern of willemite (Zn_2_SiO_4_) observed along <122>. (**e**) Electron diffraction pattern acquired from the area indicated by e in (**b**). (**f**) Calculated electron diffraction pattern of franklinite (ZnFe_2_O_4_) observed along <111>. (**g**) Element maps of the same frame as (**b**). (**h**) EDS spectrum acquired from whole CsP-3.

The examination of CsP-3 after heating at 900 °C using TEM/STEM revealed that the phase separation of CsP-3 proceeded further than that of CsP-2 because larger crystals of several dozen nanometers were distributed over CsP-3 (Fig. 5a,b). These crystals were willemite (Zn_2_SiO_4_; Fig. 5c,d) and franklinite (Fig. 5e,f), which were precipitated as a result of heating. The element maps for Si, Fe, Zn and Sn also have heterogeneous distribution because of migration and crystallization of these elements (Fig. 5g). The element map of Sn indicates that acicular cassiterite (SnO_2_) was also precipitated. Cl, K and Cs were completely undetectable in the EDS spectrum acquired from the whole CsP, which is corroborated by the result that the radioactivity of CsP-3 was almost lost by heating (Fig. 5h).

### Heating of CsPs together with soil

To mimic a CsP in soil in the environment, we heated CsP-6 in the presence of weathered granitic soil collected in Fukushima. After heating, we turned the pan upside down, and found that only soil was out of the pan and CsP-6 remained inside. The radioactivity of ^137^Cs in the CsP and soil before and after heating is shown in Table 1. Consequently, half of the original radioactivity moved from the CsP to the soil by heating. Moreover, the radioactivity of the soil was halved when the soil was divided into two parts, suggesting that the radioactivity was distributed throughout the soil. The halved soil A was scattered on an IP and kept in the dark for six days. In the readout image of the IP, many bright spots corresponding to radioactive particles appeared, although their intensities varied (Supplementary Fig. 2). Since we confirmed that no bright spots appeared when the original soil was scattered on an IP and kept in the dark for six days, the bright spots in Supplementary Fig. 2b were caused by radiocesium moved from the CsP to the soil. The radioactivity of ^137^Cs in the most intense particles indicated by the white arrow was estimated to be approximately 0.005 Bq at most by IP autoradiography. These results indicate that radiocesium in CsPs is sorbed by the surrounding soil particles when actual contaminated soil including CsPs is heated in air.

**Table 1:** Radioactivity of ^137^Cs (Bq) for CsP-6 heated with soil.

	Original	After 900 °C heating
CsP-6	1.56 ± 0.03	0.85 ± 0.07
Soil	N.D.	Halved soil A: 0.33 ± 0.02
		Halved soil B: 0.37 ± 0.01

CsP-6 in the pan after heating was transferred onto Kapton tape for the following SEM observation. Consequently, the morphology of CsP-6 did not change by heating even when it was heated together with soil as shown in Supplementary Fig. 3. Cs remained inside the CsP even after heating because it retained half of the original radioactivity (Supplementary Fig. 4). Some Fe and Zn in CsP-6 was crystallized as franklinite according to the TEM analysis (Supplementary Fig. 5a,b). The element maps of CsP-6 after heating at 900 °C showed that only Cs near the surface of the CsP disappeared (Supplementary Fig. 5c). On the contrary, Sn was concentrated at the periphery of the CsP, indicating that this CsP slightly dissolved and cassiterite was precipitated around the CsP^8^. These results are consistent with the results of other CsPs that were heated without soil, meaning that CsPs themselves show the same responses to heating regardless of the coexistence of soil.

## Discussion

It is apparent from the analysis after the heating (Fig. 3) that the loss of radiation in CsPs by heating was owing to the elimination of Cs, together with K and Cl (and probably Rb) from the silicate glass of CsPs. Probably these elements were diffused to the surface and were released from CsPs. In the case of radiocesium-contaminated soil, the addition of alkali chloride (NaCl) distinctly enhanced the release of radiocesium by heating^14,15^. Taking this phenomenon into account, it is suspected that alkali ions (mainly Cs) could be released by the existence of Cl in CsPs, presumably as a form of CsCl. However, the atomic ratio of Cl in CsPs was about half of Cs at most, and some CsPs contained a tiny amount of Cl^8^. Hence, formation of CsCl for all Cs was not possible and it was not a necessary condition for the release of Cs. One possible explanation of the lack of Cl for the release of all Cs as a form of CsCl is that Cs might be released also in the form of Cs_2_O at a sufficiently high temperature. In addition, the variance of the decrease rate of radiation (or radiocesium) among individual CsPs at a certain temperature (Fig. 1) may be related to the difference of the molar ratio of Cs to Cl in CsPs, although the difference in the radial distribution of Cs or existence/non-existence of precipitates (SnO_2_ etc.) on the surface of CsPs^8^ may be also the cause of the variance.

As mentioned above, because CsPs have a larger amount of radiocesium per volume than other radioactive materials such as soil particles sorbing radiocesium, the influence of CsPs on living things including humans and the environment is a matter of concern. However, the findings of the present study indicate that CsPs lose their radioactivity with proper heat treatment. For instance, decontamination waste from farmland and residential areas, sewage sludge, etc. are now being burned by incinerators in Fukushima^16^. If the heating temperature is sufficiently high and the duration of burning is sufficiently long, the main and fly ash probably contains no CsPs with intense radiation. Recently developed incinerators raise the burning temperature close to 900 °C to decompose dioxin^17^. Hence, we may not have to worry about the existence of CsPs in their ash. On the other hand, the temperature of forest fires and open burning of farmland may be insufficient to remove radiocesium from CsPs^18^.

The last result that radiocesium released from CsPs was sorbed to the surrounding soil is also suggestive. As shown in Supplementary Fig. 2, the radiocesium released from CsPs was sorbed in specific soil particles, probably weathered biotite according to the previous work^3,4^, but the radioactivity per particle was far lower than that of CsPs because the radiocesium released from one CsP was dispersed and distributed among a number of the soil particles. This change in the distribution and intensity of radiation can be used for the instant discrimination of CsPs in soils, and may open the way to estimating the contribution of CsPs to deposited radiocesium in the Fukushima and Kanto areas, which is not well understood at present.

## Methods

### Materials

Six CsPs named CsP-1 to 6 were collected and investigated in this study. CsP-1 was collected from plant tissue in Fukushima in 2015. CsP-2 to 6 were collected from non-woven fabric cloth laid on a vegetable field in Fukushima. The cloth had been left outside for approximately six months after the accident at the FDNPP. CsP-1 to 3 were placed onto Kapton tape as described in detail in the previous report^7^. As for CsP-4 to 6, they were separated from the non-woven fabric cloth by ultrasonic vibration in ion-exchanged water. The water was divided into several vials of 5 ml, and their radioactivity of ^137^Cs was determined using an automatic gamma counter (Cobra Quantum 5003, Packard). The aliquot of water with the highest γ-ray count rate was retained. Ion-exchanged water was added to the retained vial and the solution was then divided into several new vials, and that with the highest γ-ray count rate was selected again. These processes were repeated as many times as possible (about 30 times), and finally the CsP was isolated from the residue. The water including CsP-4 and 5 was dropped into a platinum pan of 50 μl, and the water including CsP-6 was dropped onto a plastic plate coated with carbon, and dried. CsP-1 to 3 on Kapton tape and CsP-6 on a plastic plate were identified by the following SEM observation. CsP-4 and 5 in platinum pans were presented for heating experiments.

Soil was collected from weathered granodiorite from the older type of Abukuma granitic rocks in Fukushima. We preliminarily confirmed using a germanium detector (GCW2523S, Canberra) that this soil had not been contaminated by the FDNPP accident. According to the results of powder X-ray diffraction (XRD) and X-ray fluorescence (XRF), the soil mainly consisted of quartz, K-feldspar, plagioclase, hornblende and weathered biotite, which are typical minerals in weathered granodiorite (Supplementary Fig. 6, Supplementary Table 1).

The radioactivity of ^137^Cs in the CsPs, determined by a germanium detector (GCW2523S, Canberra) is listed in Table 2. The values are corrected to those on 11 March 2011.

**Table 2:** Radioactivity of ^137^Cs.

Sample	^137^Cs (Bq)^*^
CsP-1	1.19 ± 0.03
CsP-2	0.65 ± 0.05
CsP-3	1.10 ± 0.09
CsP-4	0.50 ± 0.04
CsP-5	0.39 ± 0.03
CsP-6	1.56 ± 0.03

^*^Mean ± counting error.

### SEM

SEM observation and chemical analysis were carried out using a Hitachi S-4500 SEM equipped with a Kevex Sigma EDS.

### Heating experiments

The fragments of Kapton tape containing CsP-1 to 3 were cut out using an FIB system with a micro-sampling unit (Hitachi FB-2100), and put into a hole in a platinum plate dug using FIB as shown in Supplementary Fig. 7. The CsPs were heated at a heating rate of 10 °C/min and cooled in air using a thermogravimetric analyzer (Rigaku Thermo plus EVO TG 8120), and their radioactivity was measured after each heating and cooling operation. The radioactivity of CsP-2 and 3 was measured by IP autoradiography^4^. The platinum plate containing CsP-2 and 3 was placed in contact with an IP (BAS-MS, Fuji Film) in the dark for one hour. Readout images of the IP were produced using an IP reader (FLA-7000, Fuji Film). Another CsP was also measured as a reference material, and the radioactivity was evaluated from the IP signal intensity calibrated by that of the reference. The IP signal intensity of a CsP was calculated by integrating the intensity within a circular area 5 mm in diameter around the CsP. The radioactivity of other samples except CsP-2 and 3 were determined using a germanium detector after each heating and cooling operation.

CsP-6 was resuspended in water after SEM observation and dropped into a platinum pan. After drying, the weathered granitic soil of 30 mg was added to the pan. CsP-6 was heated at 900 °C in the presence of the soil in the same manner as other CsPs, and the radioactivity before and after heating was measured using a germanium detector.

### TEM/STEM

CsP-2, 3 and 6 after heating at 900 °C were thinned until they were electron-transparent using an FIB system. These thin specimens were preliminarily observed using a JEOL JEM-2010 TEM operated at 200 kV. Chemical analysis and elemental mapping were carried out using a JEOL JEM-2800 STEM operated at 200 kV with an X-Max^N^ 100 TLE silicon drift detector (Oxford Instruments).

### Data availability

The data that support the findings of this study are available from the corresponding author upon reasonable request.

## Electronic supplementary material

Supplementary Information
