# Supplementary material for: Loss of radioactivity in radiocesium-bearing microparticles emitted from the Fukushima Dai-ichi nuclear power plant by heating
Source: Sci Rep. 2018 Jun 26;8:9707. doi: 10.1038/s41598-018-28087-5 (PMC6018813; doi:10.1038/s41598-018-28087-5)
Supplement: Supplementary file 1 — Supplementary Information [file 41598_2018_28087_MOESM1_ESM.pdf]

Supplementary Information for

Loss of radioactivity in radiocesium-bearing microparticles emitted from the Fukushima Dai-ichi nuclear power plant by heating

Taiga Okumura[1,*], Noriko Yamaguchi[2], Terumi Dohi[3], Kazuki Iijima[3] and Toshihiro Kogure[1]

[1]Department of Earth and Planetary Science, Graduate School of Science, The University of Tokyo, 7-3-1 Hongo, Bunkyo-ku, Tokyo, 113-0033 Japan

[2]Institute for Agro-Environmental Sciences, NARO, 3-1-3 Kannondai, Tsukuba, 305-0864 Japan

[3]Fukushima Environmental Safety Center, Sector of Fukushima Research and Development, Japan Atomic Energy Agency, 10-2 Fukasaku, Miharu-machi, Tamura-gun, Fukushima, 963-7700 Japan

*Corresponding author

E-mail: okumura@eps.s.u-tokyo.ac.jp Tel: +81-3-5841-4545

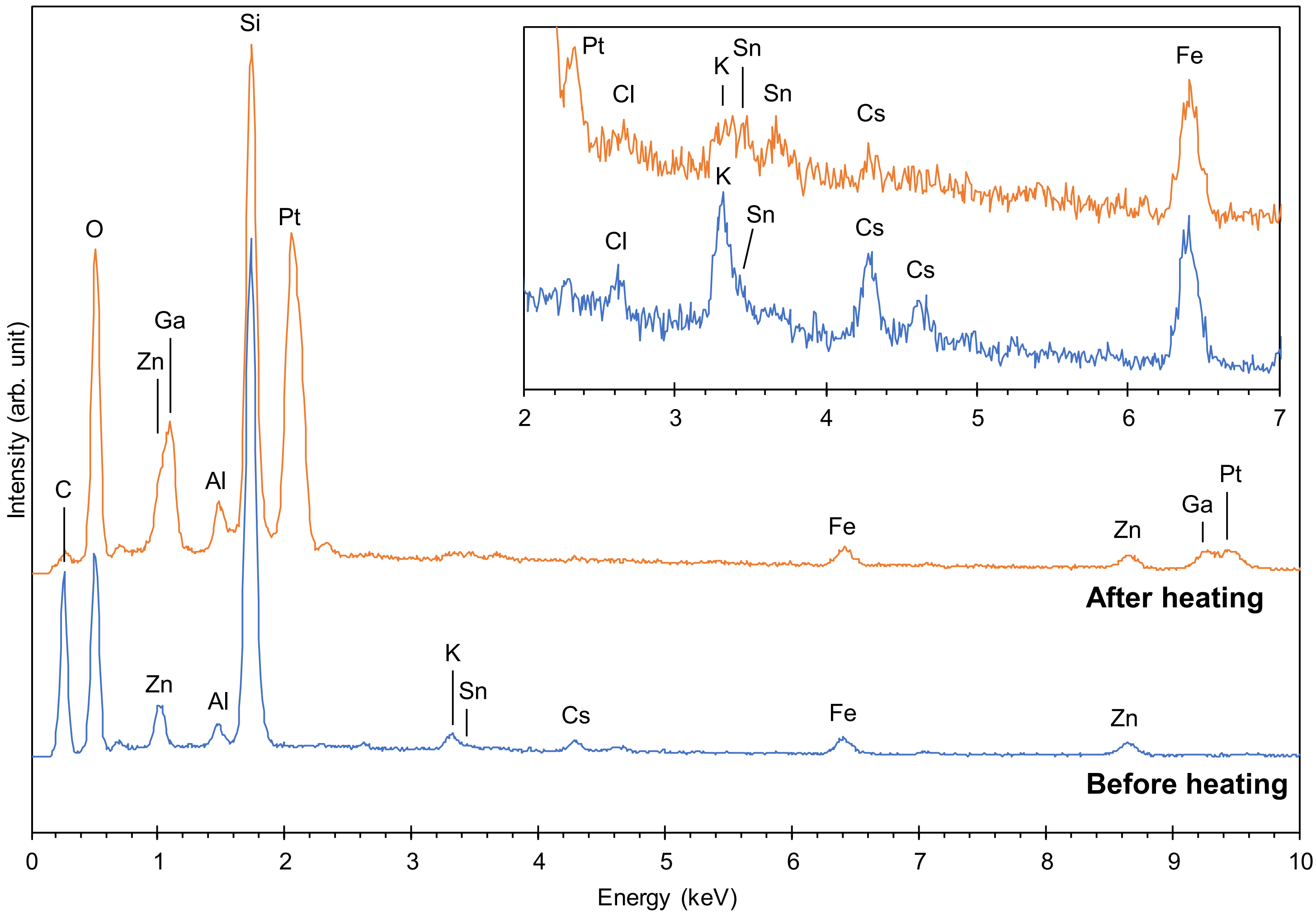

Supplementary Figure 1. Composition change of CsP-2 by heating. SEM-EDS spectra acquired from CsP-2 before and after heating at 900°C. The inset shows enlarged spectra in the range of 2–7 keV.

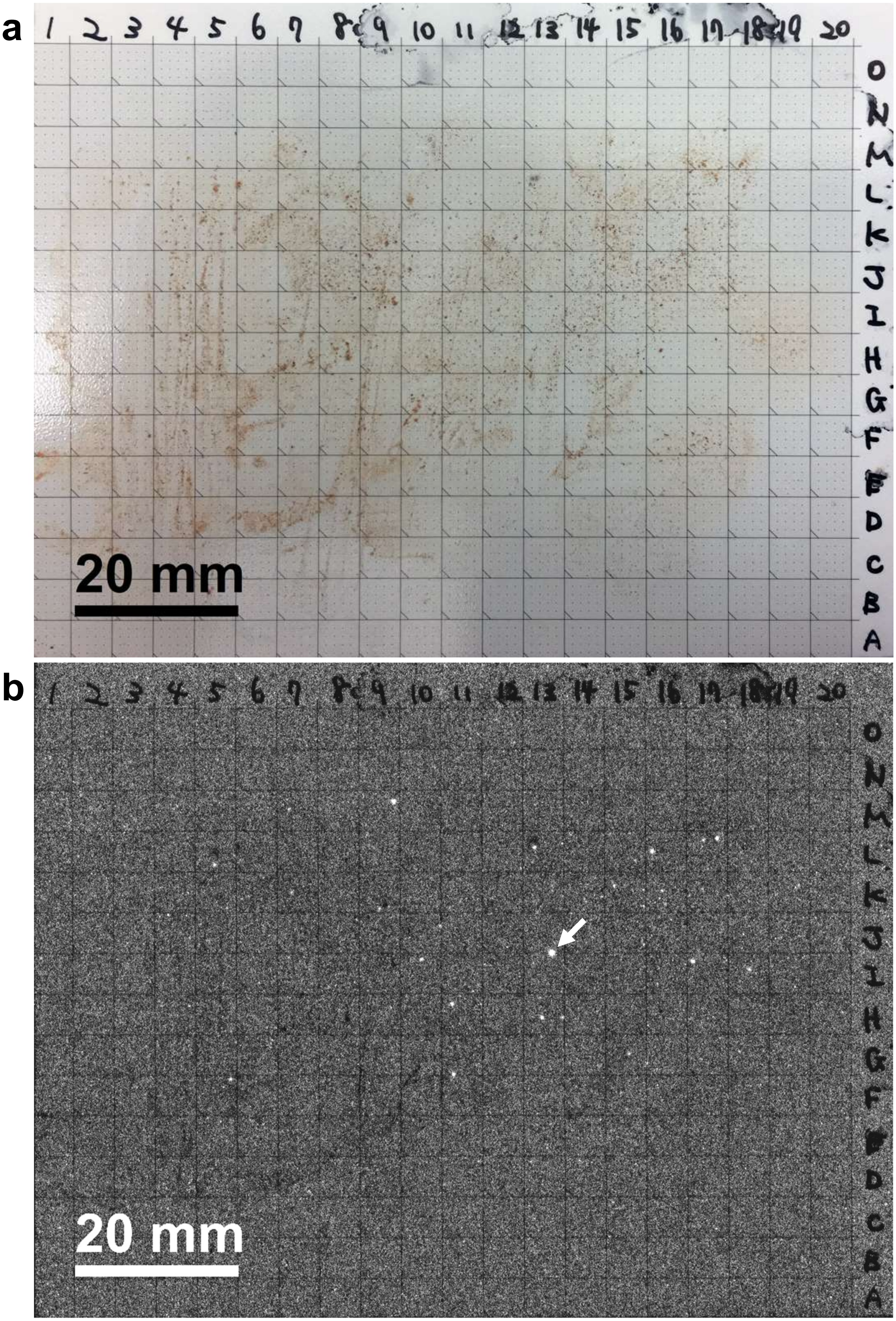

Supplementary Figure 2. IP autoradiography of soil heated together with CsP-6. (**a**) Photograph of IP on which soil heated with CsP-6 was scattered. (**b**) Readout image of IP kept in the dark for six days. The white arrow indicates the most intense particles whose radioactivity was estimated to be approximately 0.005 Bq.

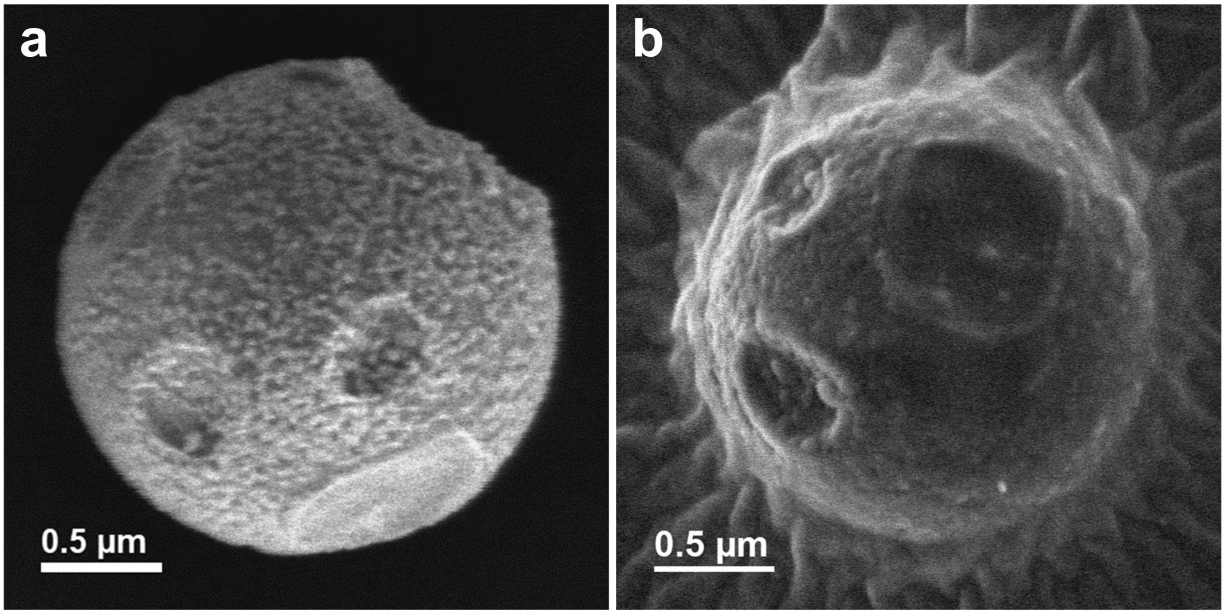

Supplementary Figure 3. Morphologies of CsP-6 before and after heating. (**a**) SE image of CsP-6 before heating on a silicon wafer. (**b**) SE image of CsP-6 after heating on Kapton tape.

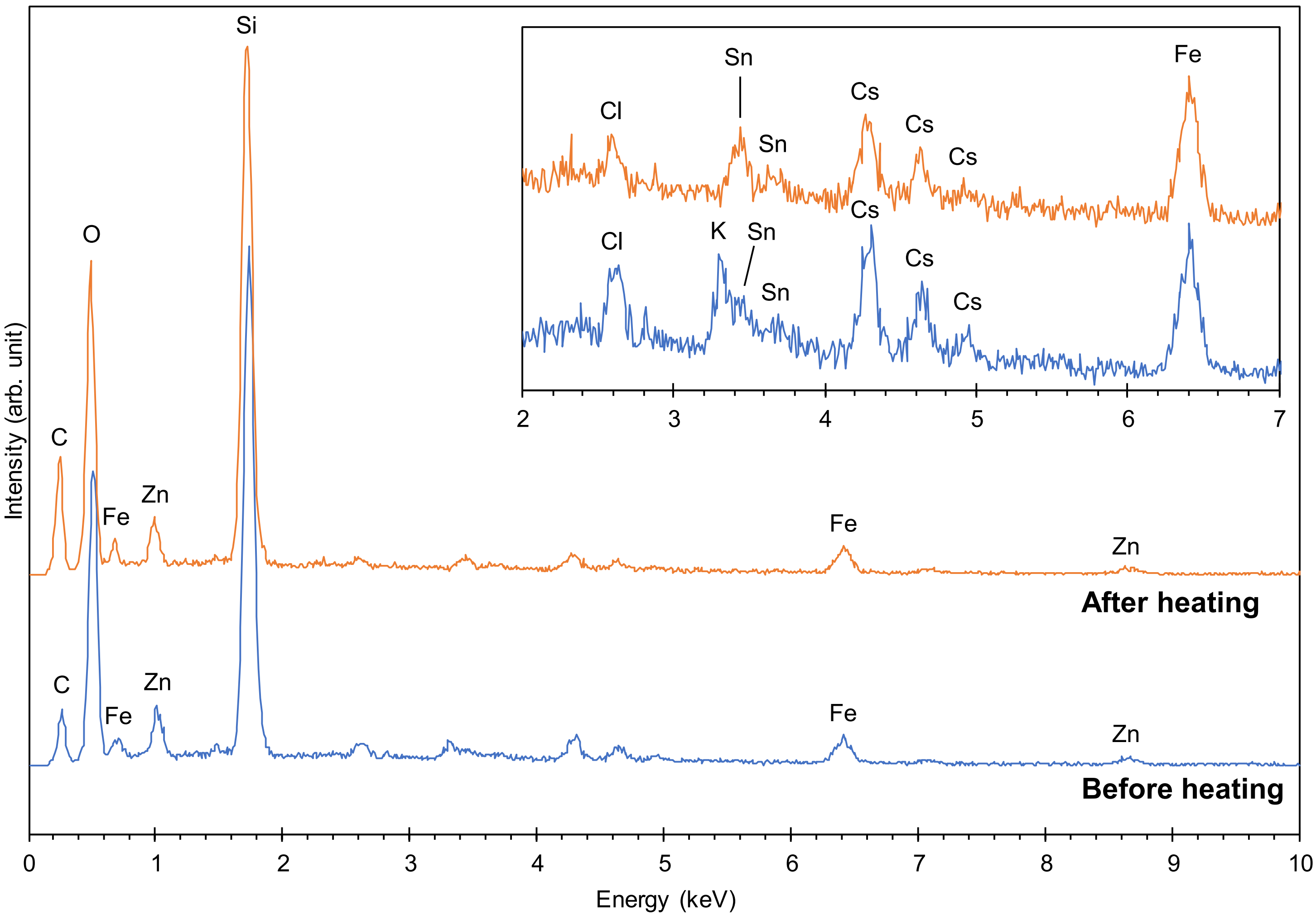

Supplementary Figure 4. Composition change of CsP-6 by heating. SEM-EDS spectra acquired from CsP-6 before and after heating at 900°C. The inset shows enlarged spectra in the range of 2–7 keV.

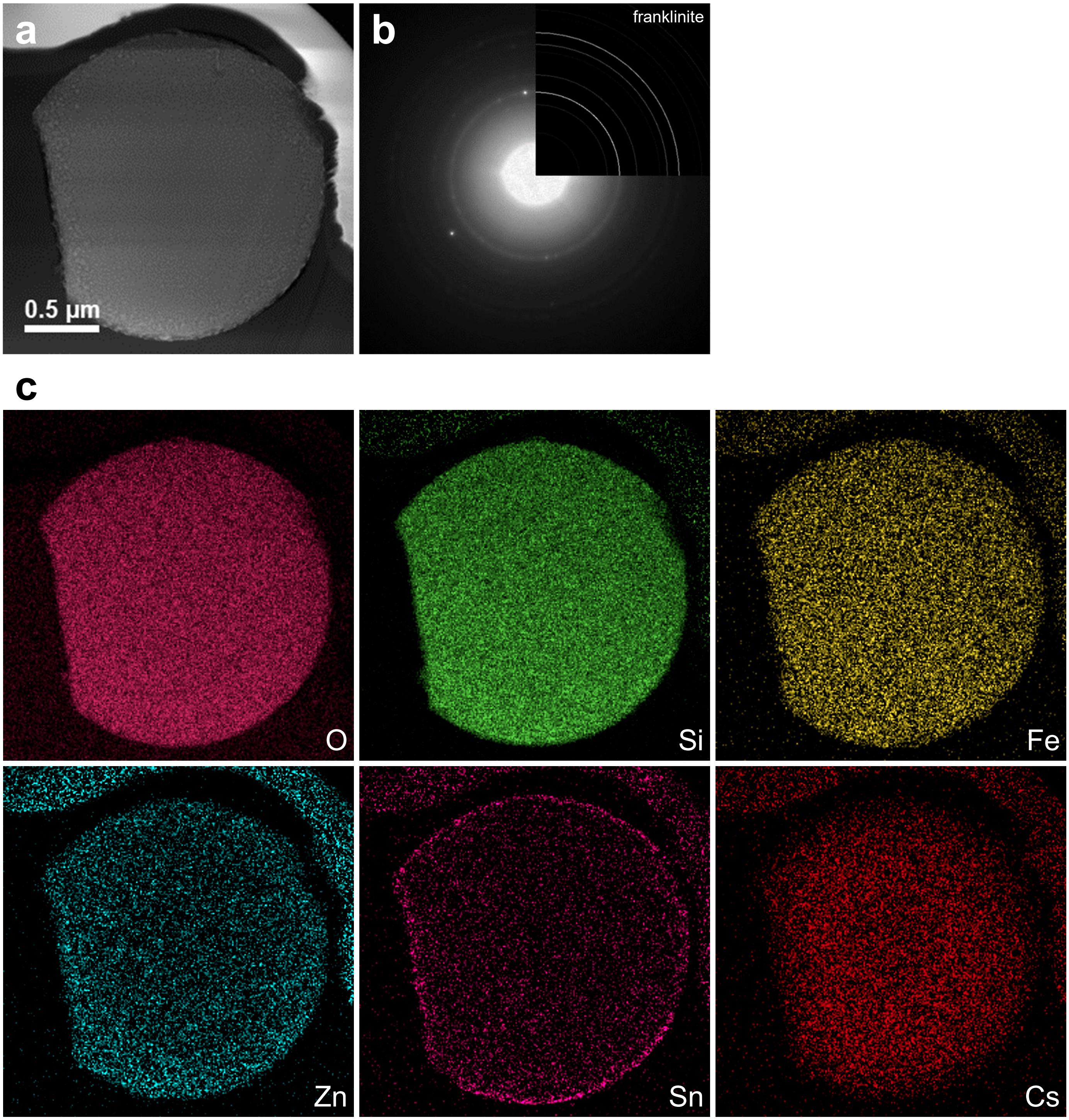

Supplementary Figure 5. TEM/STEM analyses of CsP-6 after heating at 900°C. (**a**) STEM-ADF image. (**b**) Electron diffraction pattern acquired from crystallized region of CsP-6. The inset shows the calculated Debye-Scherrer pattern of franklinite ($ZnFe_2O_4$). (**c**) Element maps of CsP-6.

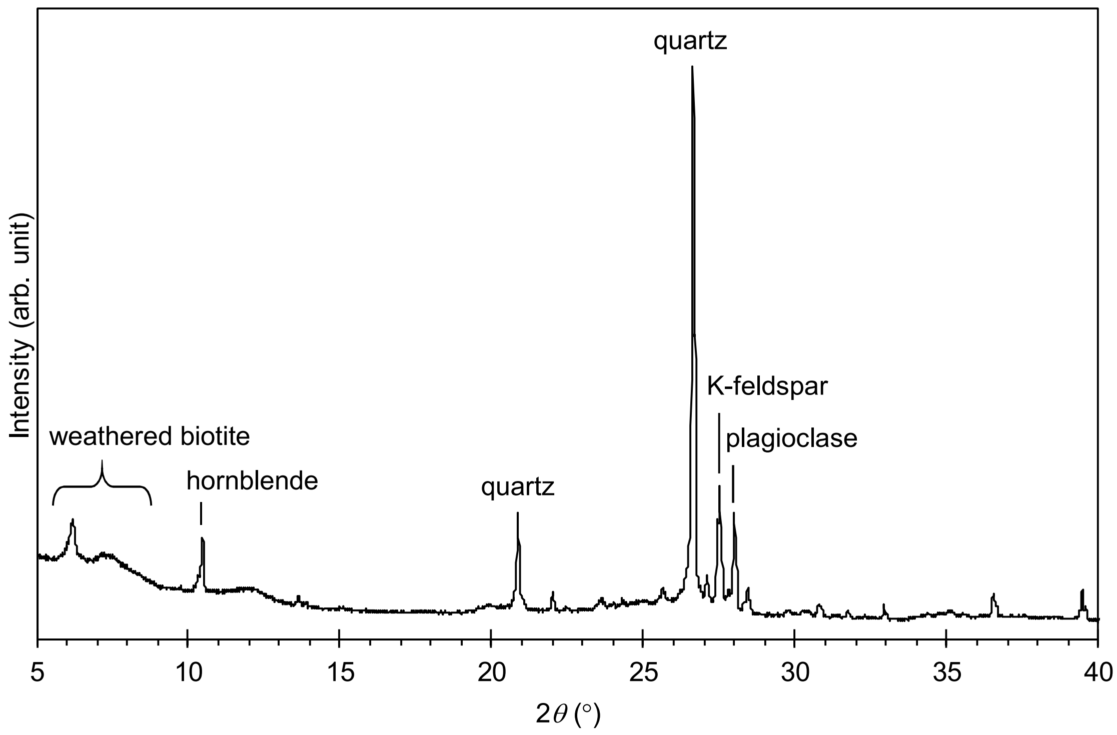

Supplementary Figure 6. Mineral phase identification of soil by XRD. XRD measurements were conducted using a Rint-Ultima$^+$ diffractometer (Rigaku) with CuKα radiation monochromated with Ni filter, and a silicon strip detector (Rigaku D/teX Ultra 2). Data was collected at a rate of 2 °/min and every 0.02°.

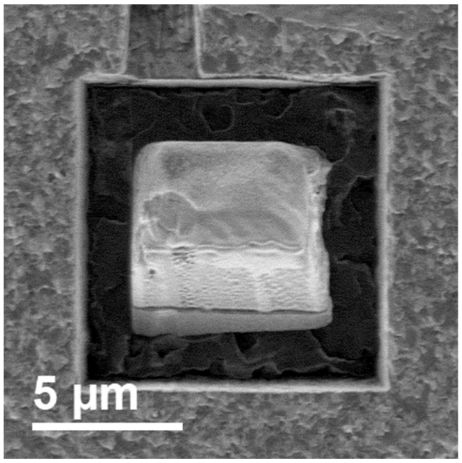

Supplementary Figure 7. Scanning ion microscope image of Kapton-tape-block containing CsP-2 in a hole dug in a platinum plate.

Supplementary Table 1. Chemical composition (wt.%) of soil determined using XRF. XRF measurements were conducted using an Axios spectrometer (PANalytical)

SiO_2	TiO_2	Al_2O_3	Fe_2O_3	MnO	MgO	CaO	Na_2O	K_2O	P_2O_5	Total
65.97	0.79	18.22	6.46	0.1	1.89	1.26	1.04	3.07	0.03	98.822
